# Active transforming growth factor-β2 in the aqueous humor of posterior polymorphous corneal dystrophy patients

**DOI:** 10.1371/journal.pone.0175509

**Published:** 2017-04-17

**Authors:** Andrea Stadnikova, Lubica Dudakova, Pavlina Skalicka, Zdenek Valenta, Martin Filipec, Katerina Jirsova

**Affiliations:** 1 Laboratory of the Biology and Pathology of the Eye, Institute of Inherited Metabolic Disorders, First Faculty of Medicine, Charles University and General University Hospital in Prague, Czech Republic; 2 Department of Ophthalmology, First Faculty of Medicine, Charles University and General University Hospital in Prague, Czech Republic; 3 Department of Medical Informatics & Biostatistics, Institute of Computer Science, Czech Academy of Sciences, Prague, Czech Republic; 4 European Eye Clinic Lexum, Prague, Czech Republic; National Eye Institute, UNITED STATES

## Abstract

**Purpose:**

Posterior polymorphous corneal dystrophy (PPCD) is characterized by abnormal proliferation of corneal endothelial cells. It was shown that TGF-β2 present in aqueous humor (AH) could help maintaining the corneal endothelium in a G1-phase-arrest state. We wanted to determine whether the levels of this protein are changed in AH of PPCD patients.

**Methods:**

We determined the concentrations of active TGF-β2 in the AH of 29 PPCD patients (42 samples) and 40 cadaver controls (44 samples) by ELISA. For data analysis the PPCD patients were divided based on either the molecular genetic cause of their disease as PPCD1 (37 samples), PPCD3 (1 sample) and PPCDx (not linked to a known PPCD loci, 4 samples) or on the presence (17 samples) or absence (25 samples) of secondary glaucoma or on whether they had undergone penetrating keratoplasty (PK, 32 samples) or repeated PK (rePK, 7 samples).

**Results:**

The level of active TGF-β2 in the AH of all PPCD patients (mean ± SD; 386.98 ± 114.88 pg/ml) in comparison to the control group (260.95 ± 112.43 pg/ml) was significantly higher (*P* = 0.0001). Compared to the control group, a significantly higher level of active TGF-β2 was found in the PPCD1 (*P* = 0.0005) and PPCDx (*P* = 0.0022) groups. Among patients the levels of active TGF-β2 were not significantly affected by gender, age, secondary glaucoma or by the progression of dystrophy when one or repeated PK were performed.

**Conclusion:**

The levels of active TGF-β2 in the AH of PPCD patients are significantly higher than control values, and thus the increased levels of TGF-β2 could be a consequence of the PPCD phenotype and can be considered as another feature characterizing this disease.

## Introduction

Posterior polymorphous corneal dystrophy (PPCD) is a bilateral disorder affecting all layers of the cornea but most severely its posterior part, i.e., the endothelium, Descemet’s membrane and the deepest stromal layers [1, 2]. PPCD is genetically heterogeneous: PPCD1 (OMIM #122000) is caused by mutations in the *OVOL2* promoter [3], PPCD2 (OMIM #609140) has been associated with mutations in *COL8A2* [4] and PPCD3 (OMIM #609141) with mutations in *ZEB1* genes [5].

PPCD affects at least 1:100,000 inhabitants in the Czech Republic and most patients carry a disease-causing founder mutation in *OVOL2* [3]. Several PPCD3 families have also been identified [6–10]. In one family linkage exclusion to the PPCD1 locus and a lack of mutations in the coding sequence of *ZEB1* and *COL8A2* suggest the possibility of the existence of a novel disease locus [10, 11].

The human corneal endothelium is a monolayer of flat hexagonal cells, which are normally arrested in the G1-phase of the cell cycle but retain their proliferative capacity [12] that may be renewed *in vivo* and *in vitro* by the disruption of cell-cell contacts and by the addition of growth factors into the anterior chamber or culture medium [13–15].

The corneal endothelial cells of PPCD patients lose their original characteristics and acquire an epithelial- or fibroblast-like phenotype [1, 16, 17]. Proliferating abnormal cells extend outwards from the cornea over the trabecular meshwork; often leading to secondary glaucoma [1, 18]. The epithelial features of these aberrant cells include abundant desmosome formation, microvilli on their apical surface and the expression of keratins [17, 19]. Descemet’s membrane becomes irregularly thickened with the presence of a posterior collagenous layer and altered collagen expression [2, 20]. It has been confirmed that the recurrence of PPCD in patients after penetrating keratoplasty (PK) is caused by the overgrowth of pathological host endothelium [21]. The precise molecular mechanisms behind the epithelialization of the corneal endothelium occurring in PPCD still remain unclear.

Transforming growth factor-beta (TGF-β) signaling is involved in almost all physiological and pathological cell behavior including regulation of immunity, differentiation, proliferation, migration, and production of the extracellular matrix [22–24]. It was shown that TGF-β2, the major isoform of the TGF-β family, is secreted into the aqueous humor (AH) as an inactive precursor by the trabecular meshwork and ciliary body [25, 26]. About 2% of the total TGF-β2 is then available in AH in its activated form [27].

Generally, activators of latent TGF-β2 include thrombospondin, matrix metalloproteinases, integrins, reactive oxygen species and/or an acidic environment [28]. Corneal endothelial cells express all three TGF-β receptors [29]. Controversy exists as to whether adult corneal endothelial cells produce TGF-β2 [30–32]. TGF-β2 has the ability to suppress S-phase entry in cultured corneal endothelial cells by blocking the degradation of p27kip1 cyclin-dependent kinase inhibitor and thus may contribute to G1-phase arrest in these cells in vivo [12, 33, 34]. In ocular disorders, the level of active TGF-β2 in human AH has been found to be both higher (primary open-angle glaucoma, keratoconus) [35, 36] and lower (uveitis, endothelial immune reactions following PK) [37, 38] than in controls.

The aim of this work was to evaluate the levels of active TGF-β2 in the AH of PPCD patients. We were also interested to determine whether the level of TGF-β2 depends on the progression of PPCD, characterized by the necessity to perform PK and/or by the concurrent presence of glaucoma.

## Material and methods

### Patients and specimen preparation

The study was approved by the Ethics Committee of the General University Hospital in Prague and Charles University (8/02 GACR, 1838/08 S-IV), and followed the tenets set out in the Declaration of Helsinki. Based on Czech legislation on specific health services (the law Act No. 372/2011 Coll.), the informed consent is not required if presented data are anonymized in the form, nevertheless, written informed consents were obtained from all subjects collected from 2008. None of the body donors were from a vulnerable population and all donors or next of kin provided written informed consent that was freely given.

The clinical diagnosis of PPCD was based on the presence of typical signs observed at slit lamp examination such as the presence of vesicular and geographical lesions associated with irregularities of the otherwise smooth posterior corneal surface and opacities at the level of Descemet’s membrane [1, 39]. The diagnosis of glaucoma was based on the results of routine clinical examination.

AH samples from PPCD patients were collected during surgery (PK, cataract surgery or glaucoma surgery) at the Department of Ophthalmology, First Faculty of Medicine and Charles University and General University Hospital in Prague between the years 1994 and 2008 and stored at -80°C until analysis. All samples were frozen within 3 hours after their collection. In total, 42 AH samples were obtained from 29 patients of Caucasian Czech origin (17 females, 12 males). Twenty two patients provided samples from one eye and seven patients from both eyes. In 4 out of the 22 patients which provided samples from one eye AH was collected repeatedly during multiple surgeries on the same eye (2 patients provided 3 samples from one eye and 2 patients provided 2 samples from one eye, i.e., in total 10 samples). The mean age ± SD at the time of surgery in the group of all PPCD patients (PPCD_all) was (46.61 ± 20.04 years).

On the basis of our previously reported molecular genetic investigations [3, 7], the patients were divided into three groups: PPCD1 (37 samples), PPCD3 (1 sample) and PPCDx (individuals from a family with an unknown molecular genetic cause not linked to any of the known PPCD loci, 4 samples). The PPCD_all patients were further grouped according to the presence or absence of secondary glaucoma, PPCD_glaucoma+ (17 samples) and PPCD_glaucoma- (25 samples), respectively, and whether or not they underwent corneal transplantation previously or on the day of AH sample collection, PPCD_PK+ (32 samples) and PPCD_PK- (10 samples), respectively. The PPCD_PK- group underwent either cataract or glaucoma surgery. Finally, patients that underwent repeated PK were grouped into PPCD_rePK (7 samples).

Control AH samples obtained from cadaveric donors were used for subsequent measurements of TGF-β2 levels [40]. In total, 44 samples were obtained from 40 donors (13 females and 27 males; mean age ± SD; 60.48 ± 12.79 years) with no history of corneal disease during the preparation of donor corneas in the Ocular Tissue Bank, General University Hospital in Prague. The samples were collected as follows: 18 samples from one eye, 18 samples pooled from the right and left eyes and 8 samples from both eyes separately. Control samples were collected between the years 2003 and 2009 within 24 hours after donor death, as performed in our previous studies on PPCD [2, 17]. The collection followed the regulations established for tissues collected for use in corneal transplantation, AH samples were not collected specifically for this study. All samples were frozen within 2 hours after their collection and stored at -80°C until analysis.

### TGF-β2 immunoassay

The concentration of active TGF-β2 (without activation of the latent form) was determined using a quantitative sandwich ELISA kit according to the manufacturer`s instructions (SB250, Quantikine®, R&D Systems, UK). Standards and samples were tested in 50 μl volume. Measurements were performed 2–4 times for each sample (based on the obtained volume of AH). The optical density was measured using an Infinite M200 spectrophotometer (Tecan, Männedorf, Switzerland).

### Statistical analysis

Descriptive statistics are reported as N, mean, standard deviation and 95% confidence interval. The normality of the distribution of the TGF-β2 values was assessed using both the Shapiro-Wilk and the Lomnicki-Jarque-Bera normality tests. *P* values of < 0.05 were considered to be statistically significant. Differences in active TGF-β2 levels were assessed using Linear Mixed-Effects Models for analyzing data involving repeated measurements on the same individuals, as implemented in the '*nlme*' package in R [41]. Random effects were associated with subjects contributing repeated measurements over time. In order to avoid bias in estimating variance components, Restricted Maximum Likelihood (REML) was used in modeling. Analysis of Variance (ANOVA) and Wald-type tests were used to assess the significance of the model parameters. The results are expressed as differences (d) in the mean active TGF-β2 values between individual groups. No valid data or data groups were arbitrarily excluded from the analysis. Low frequencies in the observed data groups (1 sample in the PPCD3 group) resulting from random sampling were left for statistical testing to provide evidence for inference. Confounding effect of age on the levels of TGF-β2 within the control group was assessed using linear regression modeling involving independent data where multiple observations per patient were replaced with the corresponding average value, and also using linear mixed model regression analysis involving the original data with possibly repeated observations per patient. Welch two-sample t-test involving independent data was used to analyze differences in age distribution between the groups of control and PPCD_all patients.

## Results

**Fig 1:**
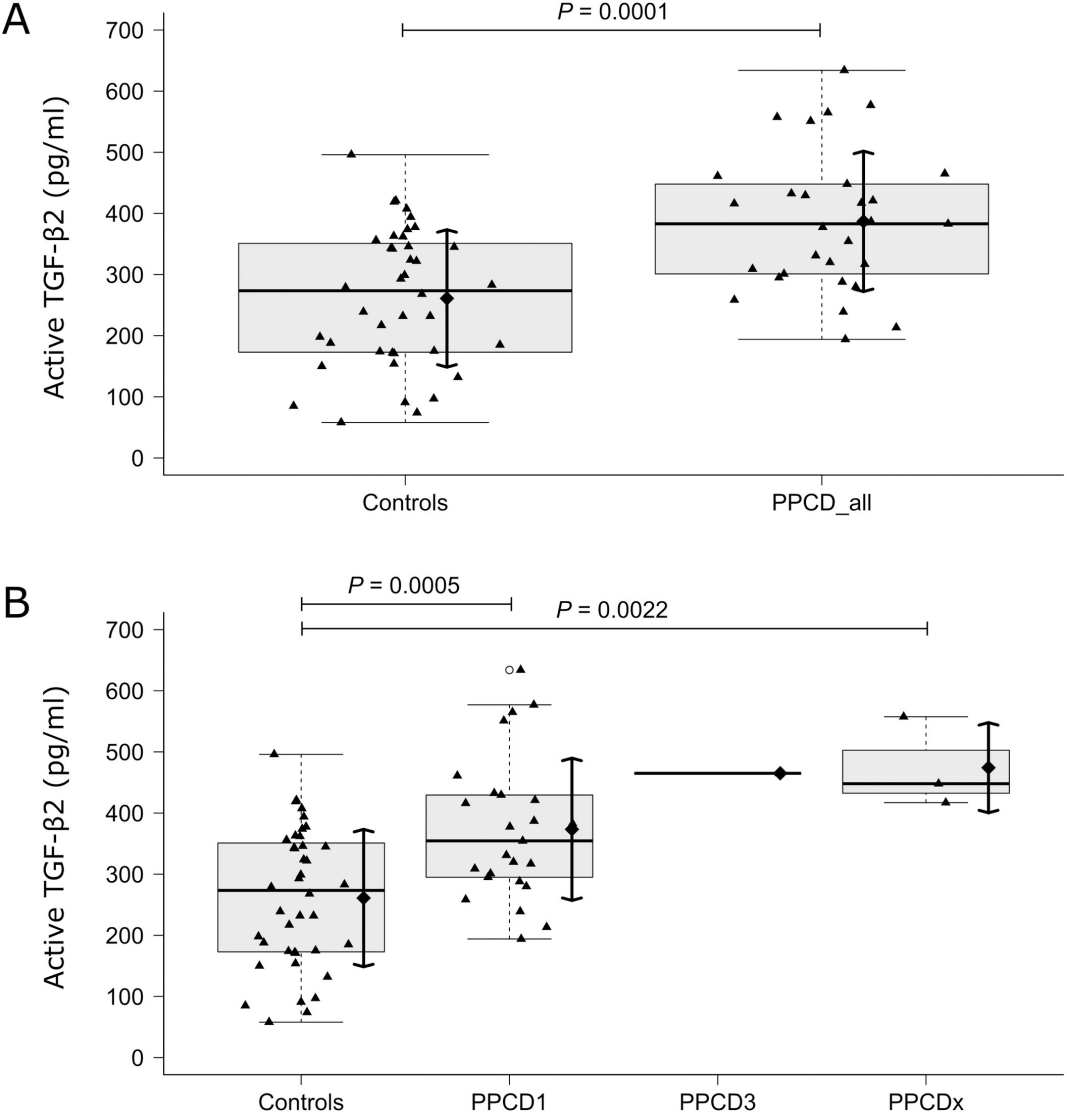
Active transforming growth factor (TGF)-β2 levels in the AH measured by ELISA. (A) Distribution of the active TGF-β2 levels in the AH of controls and all posterior polymorphous corneal dystrophy patients (PPCD_all), and (B) of controls and the groups of patients with different molecular genetic causes: PPCD1, PPCD3, and PPCDx. Multiple data points from the same patient were averaged and the average value presented as one data point. Active TGF-β2 levels are presented as data distribution and box plots enhanced with mean ± SD. Displayed *P*-values reflect statistical significance of observed differences in the mean values of TGF-β2 obtained as fixed effects in otherwise unadjusted linear mixed-effects model.

The mean levels of active TGF-β2 (mean ± SD) in the AH of the control group and all PPCD patients were 260.95 ± 112.43 pg/ml and 386.98 ± 114.88 pg/ml, respectively. All descriptive characteristics of the active TGF-β2 levels within the various target groups are presented in Table 1. The distributions of active TGF-β2 levels within the patient groups and controls are shown in Fig 1. None of the considered confounding factors, namely the age, gender, history of PK, repeated PK and glaucoma, proved to exert a statistically significant effect on the mean levels of TGF-β2. The best data summary was therefore obtained from unadjusted models that did not involve the confounding factors.

**Table 1:** Descriptive statistics.

	Active TGF-β2 [pg/ml]				
Group	N*	Mean	SD	95% LCL†	95% UCL‡
Controls	40	260.95	112.43	224.99	296.91
PPCD_all	29	386.98	114.88	343.28	430.67
PPCD1	25	373.39	116.39	325.35	421.44
PPCD3§	1	465.00	-	-	-
PPCDx	3	474.17	73.81	290.80	657.53
Controls female	13	224.46	104.89	161.08	287.84
PPCD_all female	17	378.26	86.59	333.74	422.79
Controls male	27	278.52	113.57	233.59	323.45
PPCD_all male	12	399.32	149.68	304.22	494.42
PPCD_PK+	22	378.38	108.91	330.09	426.67
PPCD_PK-	7	414.00	137.66	286.69	541.31
PPCD_rePK+	2	335.5	67.18	-268.04	939.04
PPCD_glaucoma+	11	383.98	130.21	296.51	471.46
PPCD_glaucoma-	18	388.81	108.42	334.89	442.72

^*^ Number of samples.

^*^ Multiple samples from the same patient are interpreted as an average value and presented as one sample.

^†^ Lower 95% confidence limit for the mean.

^‡^ Upper 95% confidence limit for the mean.

^§^ There is only one patient in the PPCD3 group; it is not possible to calculate descriptive statistics.

Considering all PPCD patients as one group, analysis of variance revealed significant differences in the mean levels of active TGF-β2 in the AH between the PPCD patients and controls (*P* = 0.0001). The estimated mean difference (d) in TGF-β2 values between these two groups was 120.4 pg/ml, *P* = 0.0001. Considering the three groups (i.e., PPCD1, PPCD3 and PPCDx) against the baseline (controls), analysis of variance again detected statistically significant differences in the mean levels of TGF-β2 (*P* = 0.0003). More specifically, the TGF-β2 levels in the AH were shown to be significantly higher in PPCD1 patients (d = 106.0 pg/ml, *P* = 0.0005) and in PPCDx patients (d = 216.6 pg/ml, *P* = 0.0022), as compared to the controls. The estimated mean difference in TGF-β2 values between the PPCD3 value and the controls was d = 199.2 pg/ml, *P* = 0.1040. The estimated differences in the mean TGF-β2 levels along with 95% confidence limits are shown in Table 2.

**Table 2:** Analysis of variance based on the unadjusted model for the active TGF-β2 values in individual PPCD groups.

Disease Group	Estimated difference in TGF-β2 values relative to Controls (pg/ml)	95% LCL* (pg/ml)	95% UCL^†^ (pg/ml)	*P*-value
PPCD1	106.04	48.10	163.98	0.0005
PPCD3	199.15	-42.04	440.33	0.1040
PPCDx	216.61	80.88	352.34	0.0022

* Lower 95% confidence limit for the mean.

† Upper 95% confidence limit for the mean.

Analysis of variance based on the fully adjusted model demonstrated that the presence of PPCD was the only factor that influenced the levels of active TGF-β2 statistically significantly (*P* = 0.0003). The levels were not altered statistically significantly by the presence of secondary glaucoma (*P* = 0.3067) or by the development of PPCD into a state where it was necessary to undergo PK (*P* = 0.4228) or repeated PK (*P* = 0.2879). Similarly, neither gender (*P* = 0.2138) nor age (*P* = 0.2059) proved to have influenced the levels of active TGF-β2 statistically significantly. A summary of these results is shown in Table 3.

**Table 3:** Influence of different independent factors on the levels of active TGF-β2 analyzed by analysis of variance based on the fully adjusted model.

Covariate	DF* Numerator^†^, Denominator^†^	F-statistic	*P*-value
(Intercept)	1, 64	534.4917	<0.0001
PPCD	3, 64	7.2643	0.0003
Age	1, 13	1.7728	0.2059
Gender	1, 64	1.5767	0.2138
PK	1, 13	0.6850	0.4228
Repeated PK	1, 13	1.2279	0.2879
Glaucoma	1, 13	1.1323	0.3067

* Degrees of freedom.

† Numbers representing degrees of freedom for F-statistics.

Obtained results show statistical significance between control and PPCD_all groups concerning the age (*P* = 0.002; Welch Two Sample t-test), however the effect of age within the control group is statistically insignificant (*P* = 0.675 using a linear model for independent data with averaged multiple responses per individual) and thus the age is not a significant confounder of TGF-β2 levels. Our PPCD samples were collected between the years 1994 and 2008, and thus a portion of samples is considerably older than control samples (collected between 2003 and 2009). The mean levels of active TGF-β2 (mean ± SD) in the AH of the PPCD older specimens (collected between 1994 and 2002) and younger specimens (collected between 2003 and 2008) were 368.12 ± 124.62 pg/ml and 407.18 ± 104.16 pg/ml, respectively. Difference in active TGF-β2 levels between younger and older PPCD specimens is not significant (*P* = 0.3669; Welch Two Sample t-test) and therefore the period of storage is not a confounding factor.

## Discussion

Although the precise molecular mechanisms leading to the PPCD phenotype are still unexplained, the histopathological abnormalities, particularly changes affecting the corneal endothelium, are considered to be a consequence of cell dysregulation due to a disease-causing mutation. As TGF-β2 influences cell differentiation, proliferation, migration and extracellular matrix production [22, 23], we aimed to determine whether there are changes in the levels of active TGF-β2 in the AH of PPCD patients. We found that the mean concentration of TGF-β2 in the AH of PPCD patients is significantly higher compared to that of the control group. The levels of TGF-β2 were not altered by gender, age, secondary glaucoma or the progression of dystrophy to a clinical state in which PK was necessary.

Cell-cell contact inhibition and active TGF-β2 maintain the mature corneal endothelium in a non-proliferative state (G1-phase arrest) through the activity of cyclin-dependent kinase inhibitors [34, 42]. In PPCD, aberrant cells exhibit proliferative activity and increased migration [1, 21]. Interestingly, here we have found that in PPCD the proliferation of aberrant cells is not associated with lower, but rather significantly higher TGF-β2 levels in AH compared to the control group; however, in 81% of samples the levels of TGF-β2 do not exceed 500 pg/ml, a concentration that was able to reduce the proliferation of cultured human corneal endothelial cells [43]. The responses of corneal endothelial cells in healthy endothelium and aberrant cells present in PPCD to TGF-β2 could differ. It is not clear whether the elevation of TGF-β2 is a prerequisite for the cell transition (epithelialization), or, more probably, whether higher levels of TGF-β2 may arise as a consequence of endothelial cell epithelialization. In the second case, TGF-β2 may be produced by epithelial-like cells. The expression of TGF-β2 by epithelial cells has been repeatedly confirmed, even in the corneal epithelium [44–46]. It is probable that once the primary changes (epithelialization) appear, they may escalate other, already ongoing changes characteristic of the histopathology of PPCD.

In terms of the phenotypic switch occurring in PPCD, the role of TGF-β2 should be considered due to its participation in an epithelial- or endothelial-to-mesenchymal transition and, in reverse, in a mesenchymal-to-epithelial transition [47, 48]. The corneal endothelium originates from the neuroectoderm with an important contribution from the mesoderm, but its phenotypical heterogeneity is large as corneal endothelial cells express epithelial, neuronal and mesothelial markers [17, 49–51]. In PPCD, aberrant cells exhibit increased migration and proliferative activity and gain epithelial-like (as shown morphologically and confirmed by the expression of keratin filaments) [1, 17] and fibroblast-like features (as shown morphologically) [1, 16]. Due to this large phenotypical divergence in both the original and aberrant cells, it is difficult to specify the type of transition occurring in PPCD. Recently, however, an association between PPCD1 and mutations in the promoter of *OVOL2* has been demonstrated. Mutations apparently lead to aberrant *OVOL2* expression during endothelial cell development [3]. Transcription factor OVOL2 is one of the key inducers of the mesenchymal-to-epithelial transition [52].

Numerous glaucoma studies have shown that the levels of both total and active TGF-β2 in the AH of patients with open-angle glaucoma are elevated in comparison to control specimens [35]. About 2-fold higher values were found in primary open-angle glaucoma patients compared to controls (cataract AH) [35], which is slightly more than the 1.4-fold higher value seen in our PPCD patients compared to controls. Our results did not show any statistically significant differences associated with the active TGF-β2 levels in the groups of PPCD_glaucoma+ and PPCD_glaucoma- patients. It seems that the appearance of secondary glaucoma in our PPCD patients was not directly associated with higher TGF-β2 levels in the AH. This indicates that the higher levels of TGF-β2 in PPCD patients are not related to glaucoma itself, but to changes occurring in PPCD. The reason for the higher levels of TGF-β2 (compared to control levels) found in the AH of patients with both disorders (PPCD and open-angle glaucoma) remains to be elucidated.

The mean levels of active TGF-β2 in our AH control samples obtained from cadaveric donors (260.95 ± 112.43 pg/ml) are consistent with the mean levels of active TGF-β2 measured in AH samples obtained from cataract surgery patients, which are frequently, but not exclusively, used as control specimens [35, 40]. It has been reported that the freezing and thawing of AH samples leads to a more than 4-fold increase of active TGF-β2 levels [27]. Although we cannot exclude the influence of cryopreservation on the levels of TGF-β2 measured in our samples, both control and pathological specimens were processed and stored in the same manner, and therefore the statistical differences that we found should be actual and not an artifact of cryopreservation. Due to the small volumes of the AH samples, we were not able to analyze the levels of latent TGF-β2 and thus distinguish whether the higher levels of active TGF-β2 in PPCD specimens are due to increased TGF-β2 activation or also due to increased TGF-β2 production.

In conclusion, our present study suggests that the higher levels of active TGF-β2 found in the AH of PPCD patients compared to those in control samples could be a consequence of the PPCD phenotype and can be considered as a feature characterizing this disease.

## Supporting information

S1 File Input data. (XLSX)
